# Immunomodulatory Effects of Pulmonarom^®^: In Vitro Induction of TLR and Cytokine Expression in Human Dendritic Cells

**DOI:** 10.3390/ph18060885

**Published:** 2025-06-13

**Authors:** Juan A. Hernández-Aceves, Sandra Georgina Solano-Gálvez, Arturo A. Wilkins-Rodríguez, José Delgado-Domínguez, Alberto Garcia Lozano, Carlos Cabello-Gutierrez, Lidia Flor Estela Huerta, Gladis Fragoso, Laila Gutiérrez-Kobeh, Rosalino Vázquez-López

**Affiliations:** 1 Departamento de Inmunología, Instituto de Investigaciones Biomédicas, Universidad Nacional Autónoma de México (UNAM), Cto. Escolar, C.U., Coyoacán, Mexico City 04510, Mexico; jhdzaceves@iibiomedicas.unam.mx (J.A.H.-A.); gladis@unam.mx (G.F.); 2 Unidad de Investigación UNAM-INC, División de Investigación, Facultad de Medicina, Universidad Nacional Autónoma de México, Mexico City 04510, Mexico; sandra.solanoga@anahuac.mx (S.G.S.-G.); wilkins_aar@yahoo.com.mx (A.A.W.-R.); lgutierr@unam.mx (L.G.-K.); 3 Laboratorio de Inmunoparasitología, Unidad de Medicina Experimental, Facultad de Medicina, Universidad Nacional Autónoma de México (UNAM), Mexico City 04510, Mexico; josesoterod@gmail.com; 4 Laboratorio de Inmunobiología, Departamento de Biología, Facultad de Química, Universidad Nacional Autónoma de México (UNAM), Cto. Escolar S/N, C.U., Coyoacán, Mexico City 04510, Mexico; albertogl@quimica.unam.mx; 5 Departamento de Investigación en Virología y Micología, Instituto Nacional de Enfermedades Respiratorias (INER), Calzada de Tlalpan 4502 Belisario Domínguez Tlalpan, Mexico City 14080, Mexico; carloscginer@gmail.com; 6 Laboratorio de Farmacología, Escuela Militar de Graduados en Sanidad, Universidad del Ejército y Fuerza Aérea, Secretaría de la Defensa Nacional, Mexico City 11200, Mexico; lidi7713@gmail.com; 7 Departamento de Microbiología, Centro de Investigación en Ciencias de la Salud (CICSA), Facultad de Ciencias de la Salud, Universidad Anáhuac México Norte, Huixquilucan 52786, Mexico

**Keywords:** Pulmonarom^®^, innate response, dendritic activation, toll-like-receptors, human dendritic cell, monocyte-derived dendritic cell, postbiotic, immunomodulation

## Abstract

**Background:** Bacterial lysates are known to modulate the immune response against respiratory infections. However, the effects of the commercial bacterial lysate Pulmonarom^®^ on dendritic cells—particularly human monocyte-derived dendritic cells (moDCs)—have not been studied. Additionally, limited data are available on the expression of Toll-like receptors (TLRs) and cytokines following stimulation with bacterial lysates. **Methods:** Human monocytes were isolated from buffy coats and differentiated into moDCs. Pulmonarom^®^ was lyophilized, quantified, and used to stimulate moDCs. Ultrastructural changes were evaluated using transmission electron microscopy. The expression of TLRs and selected cytokines was analyzed by flow cytometry. **Results:** Pulmonarom^®^ stimulation induced morphological changes in moDCs, including an increased number of dendrites and lysosomes. It also led to the upregulation of MHC class II molecules and TLRs 2, 3, 6, and 7. Additionally, the production of IL-4, IL-6, IL-8, and MCP-1 was significantly increased. **Conclusions:** Pulmonarom^®^ promotes moDC maturation, characterized by enhanced antigen presentation capabilities and lysosomal activity, along with increased expression of specific TLRs and cytokines. These features suggest a trained immunity phenotype in moDCs, potentially improving their ability to initiate adaptive immune responses against respiratory pathogens. To our knowledge, this is the first study to investigate the immunomodulatory effects of Pulmonarom^®^ on human moDCs, providing novel insights into its potential as an immunotherapeutic adjuvant.

## 1. Introduction

Acute respiratory tract infections represent a serious worldwide public health problem due to their high mortality and morbidity rates, complications, and sequelae, and also to economic repercussions in part due to the days of disability required by patients, representing a complex problem.

The Global Burden of Disease Study reports that in 2021, the global number of new episodes of upper respiratory tract infections was 12.8 billion for all ages in men and women. (1) Globally, the incidence rate of upper respiratory tract infections was from 162 to 484.8 per 100,000 population, with the highest incidence rate in children under 2 years old, and there were a number of episodes recorded in children aged 5 to 9 [1].

Regarding lower respiratory tract infections (LRIs), during 2024, the Global Burden of Disease Study reports that in 2021, there were 344 million cases of LRIs and 2.18 million deaths worldwide; 502,000 deaths occurred in children under 5 years of age, of which 254,000 occurred in countries with a low sociodemographic index [2].

Considering this serious problem worldwide, preventive measures have been adopted to help reduce the impact of these infections; one of them, and probably the most important, is vaccination, which has had a great impact on several diseases caused by viral and bacterial agents [3]. Although, unfortunately, not all respiratory infections can be prevented through immunization [4].

Another measure adopted to deal with respiratory tract infections is the use of bacterial lysate compounds for therapy against these infections, which have been of utility for their prevention and treatment [5]. Bacterial lysates fall under the classification of postbiotics proposed by ISAPP, which states that “A postbiotic is a preparation of inanimate microorganisms and/or their components that confer a health benefit on the host.” This definition makes no distinction between the microorganism of origin [6,7,8].

These bacterial lysates act as immunomodulators, and some reports point to their effects through the increase in specific antibodies. These properties are due to their capacity to stimulate the innate immune response. Dendritic cells are one of the main relevant cells that orchestrate the immune response [9,10]. It is important to know which effects these bacterial lysates have on this antigen-presenting cell.

Dendritic cells (DCs) are a heterogeneous cell population whose members differ in ontogeny, anatomical location, migration, cytokine secretion pattern, and immunological functions. They are located in lymphoid and non-lymphoid tissues and sense the presence of pathogens or danger molecules mainly through their pattern recognition receptors (PRRs): pathogen-associated molecular patterns (PAMPs) and damage-associated molecular patterns (DAMPs) [11]. DCs have several types of PRRs; the most well known are the Toll-like receptors (TLRs). DCs can also phagocytose microorganisms, a process that favors the migration of DCs to lymphoid organs where they perform their functions as antigen presenters to T lymphocytes, thus initiating the adaptive immune response [10]. It has also been shown that DCs participate in the modulation of the immune response towards an inflammatory response (Th1) or antibody-producing and antiparasitic response (Th2) [9] and in the regulation of cytotoxic T lymphocytes and immunological tolerance through the production of different cytokines [12].

Intranasal immunization with postbiotics induced in mice increased mRNA expression levels of CCL3, CCL4, CXCL1, CXCL2, CXCL9, and CXCL10 and moderate levels of TNF-α, as well as increased the influx of activated DCs (CD80^Hi^/CD86^Hi^) and neutrophils [13]. In a subsequent study, mice orally administered with postbiotics and subsequently infected with sublethal doses of influenza virus were shown to have lower viral loads in lung tissue and increased cytotoxic CD8^+^ T-cells, indicating heterologous protection [14]. In addition, nonspecific DC maturation was observed with the overexpression of antigen presentation markers, polyclonal B-cell activation with a significant increase in IgG serum levels, and trends toward increased influenza-specific IgA and IgG in the airways. These mice also showed little protection against secondary bacterial infections by *Klebsiella pneumoniae* and *Streptococcus pneumoniae* post-influenza infection [14]. This same finding of protection against secondary bacterial infection was reported in a later study with protection against *Streptococcus pneumoniae* [15]. Furthermore, in a recent study, it was reported that immunized mice with a postbiotic showed protection against respiratory syncytial virus, mediated by increased levels of IFNβ [16].

Although some research demonstrates the involvement of DC-TLRs in the interaction with bacterial lysates, most of these studies were performed on cell lines or with mouse bone marrow-derived DCs. In some of these studies, no more than two TLRs were analyzed (TLR2/TLR4, TLR2/TLR6, or TLR4/7), and there were some molecules of the signaling pathway, including MyD88 (an adaptor intracellular signaling molecule) [17]. The above limits the understanding of the preventive and therapeutic effects of postbiotics in human respiratory infections and which different TLR receptor profiles are involved in the dendritic cell activation compared to mice.

Pulmonarom^®^ is used for the treatment and prevention of viral and bacterial respiratory infections. It is available for sale to the public, making it an easily accessible alternative for people. However, it is important to define its effects on the immune system, with dendritic cells being of the greatest interest due to their implications in the innate and adaptive immune responses. Thus, this study aims to evaluate DCs, activation markers, immunoinflammatory cytokines, and the TLR overexpression pattern profile induced by Pulmonarom^®^.

## 2. Results

### 2.1. Pulmonarom^®^ Extract Induces Ultrastructural Morphological Changes in moDCs

**Figure 1:**
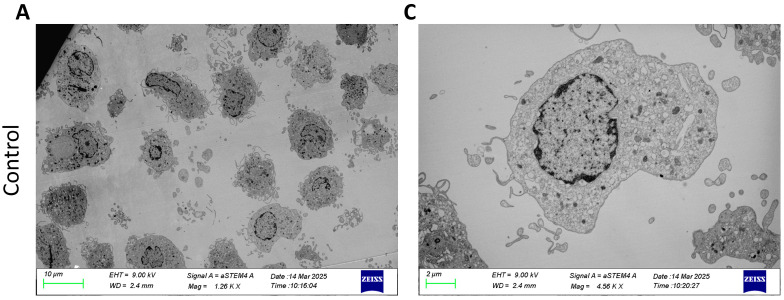

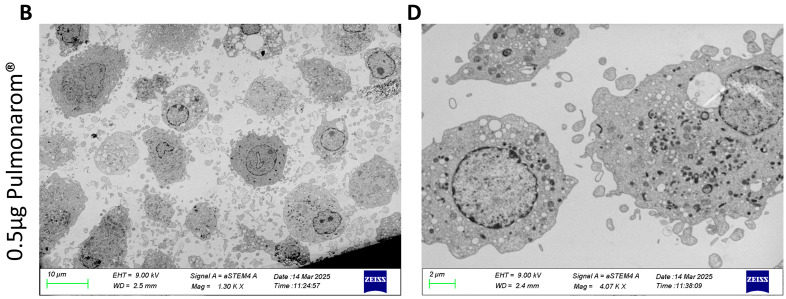
Pulmonarom^®^ simulates dendritic cells, increasing the vacuoles and vesicles. Dendritic cells were cultured in 24-well plates at a density of 1 × 10^6^ cells/mL and then fixed and prepared for transmission electron microscopy. (**A**,**C**) show non-activated human dendritic cells with a well-defined cytoplasm and few intracellular vesicles. (**B**,**D**) show human dendritic cells stimulated with 0.5 µg/mL of Pulmonarom^®^; an increase in dendrites and intracellular vesicles is observed, suggesting cell activation and an enhancement in proteolytic capacity. Scale bars: 10 µm (**A**,**B**) and 2 µm (**C**,**D**).

Monocyte-derived dendritic cells (moDCs) were cultured with different concentrations of Pulmonaron^®^ (from 0.01 to 0.5 µg/well). For the MET study, moDCs were analyzed without stimulation and stimulated with 0.5 mg of Pulmonarom^®^. As shown in Figure 1, Pulmonaron^®^ induced ultrastructural morphological changes of moDCs, as observed in transmission electron microscopy (TEM).

### 2.2. Pulmonarom^®^ Extract Increases Class II Histocompatibility Molecules in Dendritic Cells

**Figure 2:**
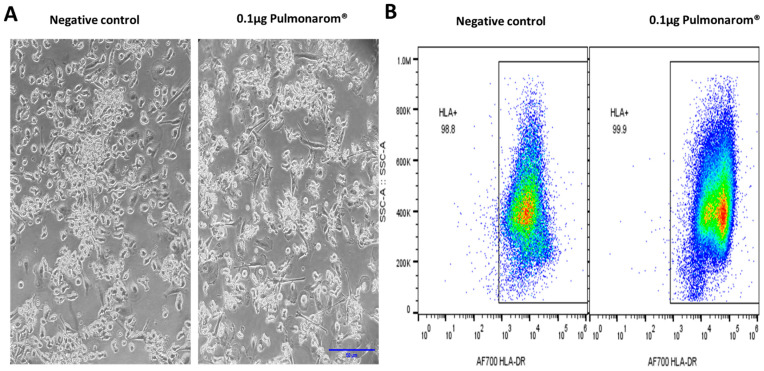

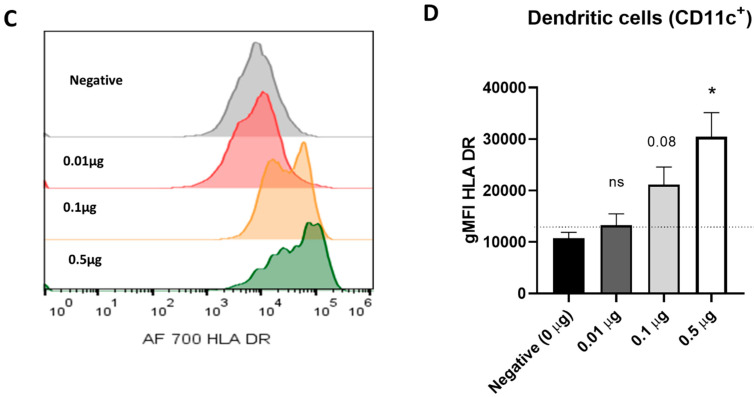
Pulmonarom^®^ increases the expression of MHC-II molecules in moDCs. (**A**) Micrographs of cultured moDCs depicting an increase in adherent cells; scale bar 50 µm in blue. (**B**) Representative dot plots from cell cultures; left and right images correspond to non-stimulated and Pulmonarom^®^-stimulated cells, respectively. An increase in HLA-DR expression is observed after stimulation with Pulmonarom^®^. (**C**) Representative histograms of HLA-DR expression. The negative control was treated only with culture medium, while the rest of the samples were stimulated with Pulmonarom^®^ at different concentrations. An increase in the expression of the HLA-DR molecule is observed. (**D**) Quantification of HLA-DR expression using the geometric mean fluorescence intensity (gMFI), dotted line indicates the maximum value in negative control. The normality of the data was assessed with a Shapiro–Wilk test. * *p* < 0.05; ns: no statistical differences. ANOVA–Dunnett was used to compare the groups, finding only a significant difference in the 0.5 µg group. n = 5 per group.

Considering the morphological changes induced by Pulmonaron^®^ over moDCs, we wondered if these changes corresponded to an activation of the cells. Figure 2 shows that Pulmonaron^®^ at a dose of 0.5 µg/well induces a significant increase in class II histocompatibility molecules HLA-DR, which is related to their activation.

### 2.3. Pulmonarom^®^ Extract Increases the Expression of TLRs 2, 3, 6, and 7

**Figure 3:**
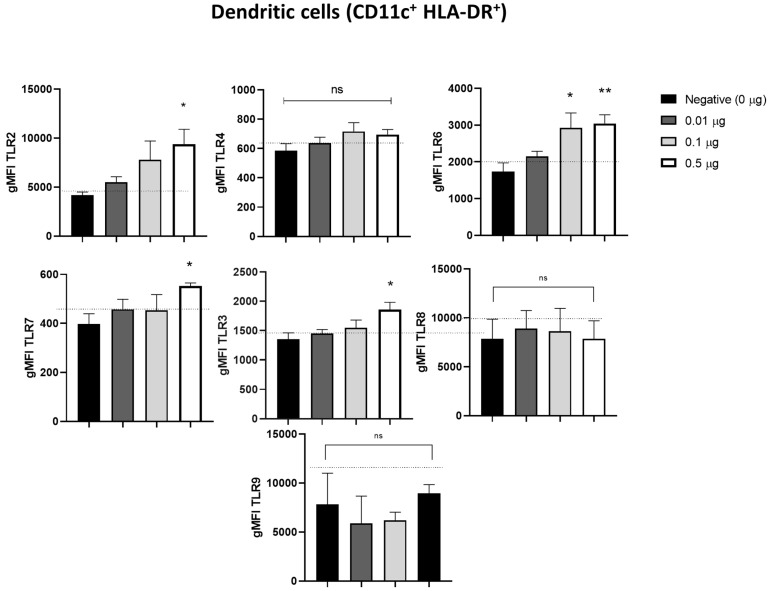
Pulmonarom^®^ increases the expression of TLRs 2, 6, 7, and 3 in dendritic cells. Dendritic cells cultured in 24-well plates at a density of 1 × 10^6^ cells/mL were stimulated for 24 h with Pulmonarom^®^ (from 0.01 to 0.50 µg/mL). After stimulation, the cells were identified by flow cytometry using the markers CD11c^+^HLA-DR^+^, and their TLR expression was evaluated. Dotted lines indicate the maximum values in negative controls. The main changes were observed in TLR6, TLR7, and TLR3. However, there was also a significant increase in TLR2. The normality of the data was evaluated with a Shapiro–Wilk test. * *p* < 0.05 and ** *p* < 0.01; ns: no statistical differences. ANOVA–Dunnett was used to compare the groups. n = 5 per group.

It has been reported that activated dendritic cells can increase the expression of TLRs, and we studied if *Pulmonarom^®^* exerts these effects. As shown in Figure 3, moDCs stimulated in vitro with Pulmonarom^®^ increased the expression of TLR2, TLR3, TLR6, and TLR7. This increase was more evident with a Pulmonarom^®^ concentration of 0.5 µg/well. For TLR6, a high increase was detected at 0.1 and 0.5 µg/well of Pulmonarom^®^. No significant effects were observed in the overexpression of TLR4, TLR8, and TLR9.

### 2.4. Pulmonarom^®^ Increases the Production of IL-4, IL-6, IL-8, and MCP-1

**Figure 4:**
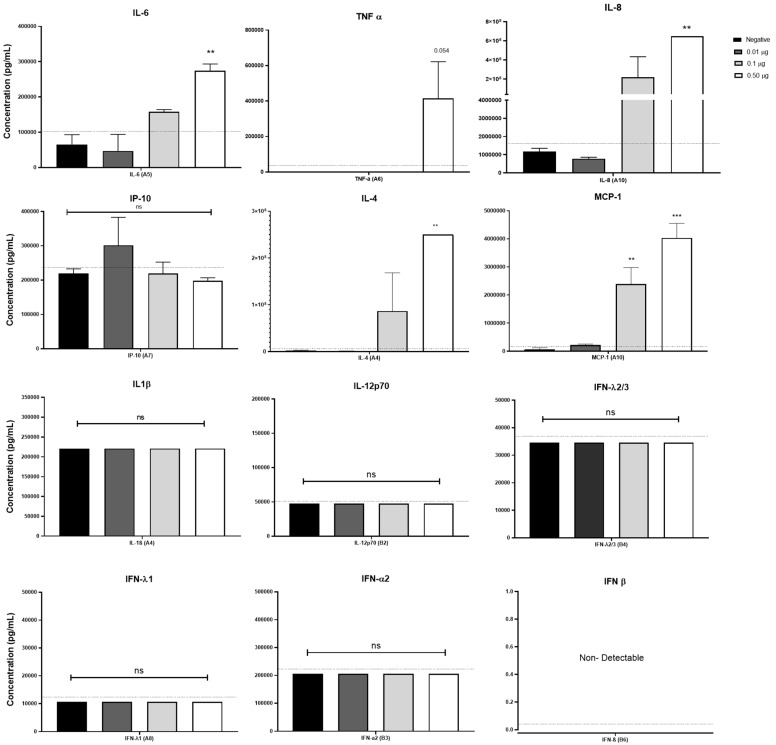
Pulmonarom^®^ increases the production of innate response cytokines. Monocyte-derived dendritic cells (moDCs) were cultured for 24 h with different doses of Pulmonarom^®^ (from 0.01 to 0.50 µg/mL) to subsequently quantify the cytokines present in the culture supernatants. The results show a significant increase in the production of IL-6, IL-8, IL-4, and MCP-1. Mean ± standard deviation (SD) was plotted. Dotted lines indicate the maximum values in negative controls. Data normality was assessed with a Shapiro–Wilk test. ANOVA–Dunnett was used to compare the groups. ** *p* < 0.01, *** *p* < 0.001; ns: no statistical differences. n = 5 per group.

It is well known that some bacterial lysates can induce the expression of inflammatory cytokines [18,19]. Considering that Pulmonarom^®^ was able to induce the overexpression of some TLRs and class II MHC molecules, this activation profile may be probably accompanied by the induction of inflammatory cytokines. As shown in Figure 4, Pulmonarom^®^ was capable of increasing IL-6, IL-8, IL-4, and MCP-1 by moDCs after 24 h of stimuli. No increase in the most inflammatory cytokines (IL-1β and TNF-α) and of type I IFNs (alpha, beta, and lambda) was detected.

## 3. Discussion

Dendritic cells play a crucial role in linking innate and adaptive immune responses to deal with pathogens. During viral and bacterial infections, the proper functioning of dendritic cells is essential for the elimination of pathogens by recognizing pathogen-associated molecular patterns (PAMPs) [19] that interact with pattern recognition receptors (PRRs) on their membrane, such as Toll-like receptors (TLRs). DC activation, through various PAMPs, enhances the maturation of phagosomes and increases the phagocytosis of pathogens [20].

Therefore, DC activation is pivotal for an adequate activation of the immune system. Additionally, DC activation triggers the production of cytokines, which boost the defense against pathogens, including pro-inflammatory cytokines, like TNFα [21], or defensins, such as β-defensin [22]. This activation also increases adhesion molecules in the endothelium, leading to greater cellular infiltration in the lesion area, thereby aiding in the elimination of various pathogens [23].

Several approaches to prevent and deal with respiratory infections have been applied worldwide, and since the 1950s, bacterial lysates have been used to reduce the incidence of recurrent respiratory tract infections [24]. Since then, commercially available bacterial lysates, including Ismigen, LW 50020, OM-85, Lantigen B, and Pulmonarom^®^, have been prescribed in children and adults.

Pulmonarom^®^ is a commercial bacterial lysate that has been marketed in Mexico for over 90 years, with registration number 13485 SSA IV. It contains 6 × 10^8^ CFUs of each of the following bacterial species: *Haemophilus influenzae*, *Staphylococcus aureus*, *Moraxella catarrhalis*, *Klebsiella pneumoniae*, *Streptococcus pneumoniae*, *Streptococcus pyogenes*, *Streptococcus agalactiae*, *Streptococcus dysgalactiae*, and *Streptococcus anginosus*. Pulmonarom^®^ is administered orally in one 3 mL ampoule daily for ten days. After thirty days, the cycle begins with one ampoule daily for another ten days. There is evidence that postbiotic lysates exert local effects in the gut, especially on mucosal immune tissue (e.g., dendritic cells, M cells, macrophages), leading to immune modulation at distant sites, such as the respiratory tract [25,26,27,28,29]. This supports the idea of the oral administration of bacterial lysates, such as Pulmonarom^®^, and their effect on the respiratory tract.

Pulmonarom^®^ is indicated for the prophylaxis of respiratory infections during endemic periods; and, since it reduces the duration of treatment for respiratory infections, it is also indicated as an adjuvant in these treatments. Pulmonarom^®^ is also indicated for nonspecific respiratory tract conditions that present without fever but with local irritation, congestion, and inflammation of the airways related to environmental pollutants. Pulmonarom^®^ is also indicated for the treatment of recurrent respiratory infections.

Despite the broad employment of Pulmonarom^®^ in Mexico, no studies have studied the mechanisms through which it can exert its therapeutic and preventive functions against respiratory tract infections. Thus, we explore the effects of this commercial postbiotic on dendritic cell activation, considering the relevant role of these cells in modulating the innate and adaptive immune response.

In this study, we found that Pulmonarom^®^ is capable of increasing antigen presentation molecules on the surface of moDCs (Figure 2). These changes are accompanied by morphological alterations in moDC cultures, indicating the maturation and activation of dendritic cells induced by postbiotics [30,31]. These effects on DCs could imply an improved potential to mobilize lymph nodes and present antigens to initiate an effective adaptive immune response.

Transmission electron microscopy (TEM) analysis revealed ultrastructural changes that correlate with moDC maturation. Pulmonarom^®^-stimulated moDCs showed a greater number of dendrites, which were observed as a large number of semicircular bodies under the TEM cross-section, as well as an increase in the number of lysosomes (Figure 1).

In immature DCs, internalized antigens are slowly degraded and inefficiently utilized for peptide loading by MHC-II; however, during maturation, the vacuolar proton pump is activated, which enhances lysosomal acidification and antigen proteolysis, facilitating the efficient formation of peptide–MHC class II complexes. Therefore, it is suggested that lysosomal function in DCs may play an important role in the processing of internalized antigens [32]. The importance of lysosomes in DC maturation, as well as their migration and chemotaxis capacity, is regulated by transient receptor potential melastatin 2 (TRPM2)-mediated lysosomal Ca^2+^ release [33]. When an appropriate DC activation occurred, the number of lysosomes also increased, as observed in moDCs stimulated with Pulmonarom^®^.

While immature DC migration follows a random, homing pattern, mature DC migration exhibits a continuous, directional pattern toward lymph nodes for antigen presentation to Th cells. This pattern of mature DC migration is regulated by lysosomal calcium levels. Although much is unknown about intracellular signaling in DC maturation, lysosomal signaling is crucial for this process. When DCs detect foreign particles, intracellular signaling is initiated, leading to lysosomal calcium being released by the ionic channel TRPML1 (transient receptor potential cation channel, mucolipin subfamily, member 1), which activates the actin-based motor protein myosin II, located at the posterior end of the cell, promoting rapid and directional migration. Lysosomal calcium also activates the transcription factor EB (TFEB), which translocates to the nucleus to maintain TRPML1 expression, thus sustaining DC migration [34]. The TRPML1-TFEB interaction negatively regulates macropinocytosis upon detection of microorganisms by DCs, thus promoting that the DC is not “distracted” by capturing other antigens during its migration [34].

In summary, to meet the dynamic functional requirements during DC maturation, extensive remodeling of lysosomal compartments occurs. These changes are necessary for lysosomal calcium efflux and the contraction of Actomyosin for DC motility and migration. Furthermore, increased lysosomal membrane permeability participates in inflammasome activation. Lysosomal signaling collaborates with intracellular signaling for antigen presentation and other immune response mechanisms [35].

Bacterial lysates can be recognized by various cellular receptors, including TCRs (T-cell receptors), BCRs (B-cell receptors), TLRs (Toll-like receptors), and TNFRs (TNFα receptor superfamily). Following such recognition, various intracellular signaling cascades are initiated, leading to different biological response scenarios. One of the signaling pathways activated after TLR recognition involves activated B-cell kappa (NF-κB) and/or mitogen-activated protein kinase (MAPK). The participation of NF-κB and/or MPAK is associated with various biological responses; for example, their participation in the cell cycle, either leading to cell death or survival, as well as their involvement in inflammation and the immune response [36,37,38,39].

When evaluating the TLRs, we found changes predominantly in the TLR2, TLR3, TLR6, and TLR7 receptors, which recognize bacterial and viral patterns. TLR2 recognizes peptidoglycan, which is present in higher concentrations in Gram-positive bacteria, such as *Staphylococcus* spp. and *Streptococcus* sp., both of which cause multiple respiratory infections [23]. Similarly, TLR6 recognizes diacyl peptides, which are also primarily expressed in Gram-positive bacteria. Consequently, the increased expression of these TLRs enhances the recognition of bacteria that mainly affect the respiratory tract.

Alyanakian et al. demonstrated that other commercial lysates stimulated mouse dendritic cells and induced TGF-β production by splenocytes in a TLR2, TLR4, and MyD88-dependent manner [40]. In this work, we also found an increase in receptors, such as TLR2 and TLR6, which can recognize nonspecific components of the bacterial membrane (Figure 2), as well as TLR3 and TLR7, which could enhance the antiviral response. In 2017, Dang et al. demonstrated that other commercial lysates induced the production of interferon-β through MyD88 and TRIF (another TLR adapter molecule) in mouse bone marrow-derived DCs [41].

Although we did not observe changes in interferon molecules, two factors were different: first, the use of this commercial lysate was in cells from C57BL/6 mice characterized by inflammatory responses [42], and second, the concentration of this commercial lysate used was 40 times higher than Pulmonarom in this study (Figure 4).

In addition, it has been reported that the participation of TLR6, TLR2, and TLR7 actively participates against some viral infections, such as the SARS-CoV-2 virus, which is recognized by TLR2 and subsequently by TLR7 for an effective antiviral response [43]. Another example is the role of TLR6 and TLR2 in the antiviral response against respiratory viral disease (RSV), where infection increases TLR2 expression and the absence of TLR2 or TLR6 leads to a higher viral load and lower production of pro-inflammatory cytokines [44]. In this work, we found an increase in TLR2, TLR6, and TLR7 (Figure 3) that could favor the recognition of some common viruses in the respiratory tract.

On the other hand, Pulmonarom ^®^ increased the expression of TLR3. This receptor is a specialized receptor for the recognition of dsRNA, so it is of great importance in viral infections. It has been reported that its participation is critical for the response against RSV in epithelial cells of the respiratory tract [45]. In addition, TLR3 is involved in the recognition of the influenza virus [46]. Therefore, observing an increase in this receptor could increase the innate immune response and induce type I interferons in the presence of ligands to this receptor [47]. However, in cases of prolonged activation, it can cause dysfunction in the immune response [48]. This is a very interesting result compared to other commercial lysates, which do not report increased TLR3 expression in their published models. Being such a ubiquitous receptor for virus recognition, it could be an advantage in functionality and coverage against various viruses that are recognized by TLR3.

The above indicates that the increase in TLRs by Pulmonarom^®^ could impact a better response against viruses and bacteria by increasing the activation of dendritic cells and their expression of TLRs; however, this has to be tested in vivo models. It should be noted that the safety of the extract in the viability of the cells is an important point since it does not represent a risk for dendritic cells (Supplementary Figure S3).

Pulmonarom^®^ induced the production of IL-6, IL-8, and MCP-1, indicating that it is activating NF-κB in dendritic cells, a result similar to that obtained using OM-85 BV in vitro. Using a mouse macrophage cell line, they demonstrated that the production of IL-1β, IL-6, and TNF-α is induced through activation mediated by the TLR4 and TLR2 signaling pathway through ERK1/2 and NF-κB [49]. In this work we did not explore the signal pathway; this is an important point to research in the future to know the molecular mechanism of Pulmonarom^®^.

IL-6 and IL-8 are two cytokines that have been widely studied in the inflammatory response.

On the one hand, IL-6 is an interleukin with pleiotropic functions; in some contexts, it can increase the immune response by increasing the specificity of CD8 T lymphocytes [50], and in others, it can promote infection and sometimes generate exacerbated inflammation, as occurs in the context of SARS-CoV-2 [51]. The participation of this cytokine at the beginning of an infection could be beneficial by increasing the phagocytic capacity of neutrophils and macrophages [46,47]. However, it is an important result that points out the importance of using in vivo models in future studies to know the role that IL-6 may have in an infectious or inflammatory context in the presence of Pulmonarom^®^.

IL-8 is a chemotactic cytokine for neutrophils, which are cells of the immune system with great phagocytic, inflammatory, and immunological capacity. They are crucial for limiting the replication of viruses in early stages [52], and even if antibodies existed previously, they can increase the elimination of viruses through antibody-mediated phagocytosis, as occurs in antibodies against hemagglutinin of the influenza A virus [53]. However, IL-8 also participates in the adaptive immune response since the ability to allow the recruitment of effector memory T lymphocytes with greater cytotoxic activity has been reported [54]. However, it is important to note the duality that IL-8 presents in inflammatory contexts since it has also been seen as associated with adults infected with the influenza virus, which is negatively correlated with the production of antibodies against the virus [55]. This is probably because IL-8 can recruit myeloid suppressor cells (MDSCs), which are increased in adult stages [56,57]. This highlights the importance of conducting in vivo studies to evaluate the effect on young and old mice and thus understand the implications that the treatment could have on people susceptible to viral infections.

IL-4 is a cytokine considered anti-inflammatory by the regulation of Th1 response and a reduction in classical inflammatory profile, and it plays a key role in the immune system by the promotion of the maturation of B-cells and the production of antibodies [58]. However, it is necessary to evaluate Pulmonarom^®^ in an in vivo model to understand how the changes work in all of these cytokines and chemokines.

The immune response needs to have the ability to recognize and present antigens to develop an effective immune response. In this work, we evaluate the ability of Pulmonarom to induce activation in dendritic cells; however, the immune response is a complex process in the organism.

In summary, the increased expression of HLA-DR molecules, as well as TLR2, TLR3, TLR6, and TLR7 receptors, accompanied by the increased production of cytokines IL-8, MCP-1, IL-6, and IL-4, could be indicative of a potential training process in moDCs following their activation with the bacterial lysate Pulmonarom^®^ [59,60]. This training may explain the protective effect of this bacterial lysate against certain respiratory infections. However, further studies are required to evaluate the presence of additional markers that are considered classic signs of immune training, signal pathways, and specific alterations in the energy metabolism of moDCs [61], as well as challenges in biological models.

## 4. Materials and Methods

### 4.1. Monocyte-Derived Dendritic Cell Obtention

Monocyte-derived dendritic cells (moDCs) were differentiated from CD14^+^ monocytes as described previously [62,63]. Peripheral blood mononuclear cells (PBMCs) were purified from buffy coats from healthy donors, which were kindly supplied by the blood bank of the National Institute of Cardiology “Ignacio Chávez”. The handling of blood samples was performed according to the Declaration of Helsinki; donors signed informed consent forms, and the local scientific and ethics committees approved the protocol. PBMCs were separated by gradient centrifugation with Histopaque-1077 (Sigma-Aldrich; Merck KGaA, Darmstadt, Germany). For this, buffy coats were diluted 1:2 WITH D-PBS to form the gradient and centrifuged at 400× *g* at 24 °C for 45 min. Then, the layer containing mononuclear cells WAS recovered, and the cells were washed three times with D-PBS at 300× *g* at 4 °C for 10 min. Afterward, mononuclear cells were blocked with BSA contained in a blocking buffer (D-PBS, 2 mM EDTA, and 0.5% BSA cell culture grade) and incubated at 4 °C for 15 min. Immediately, mononuclear cells were incubated with anti-CD14 magnetic microbeads (Miltenyi Biotec, Bergisch Gladbach, Germany) for 20 min at 4 °C and washed with D-PBS. Cells marked with anti-CD14 were separated through an LS magnetic column coupled to a magnet (Miltenyi Biotec, Bergisch Gladbach, Germany). The column was washed three times with 3 mL of D-PBS to eliminate CD14^−^ cells and then it was removed from the magnetic field, allowing the recovery of CD14^+^ monocytes using a plunger and D-PBS. The monocytes were then counted and seeded in RPMI-1640 medium containing stable glutamine (Biowest, Riverside, MO, USA) supplemented with 10% fetal bovine serum (FBS), 100 U/mL penicillin, 100 µg/mL streptomycin, and 50 µM 2-mercaptoethanol at pH 7.2 (referred to as R-10 medium). Cells were plated at a density of 1 × 10^6^ cells/mL in 24-well tissue culture-treated plates, with a final volume of 1 mL per well. The following day, fresh R-10 medium enriched with 500 U/mL granulocyte macrophage–colony-stimulating factor (GM-CSF) and 1000 U/mL IL-4 (BD Biosciences, Franklin Lakes, NJ, USA) was used to replace 50% of the medium of each well to promote the differentiation of CD14^+^ monocytes to immature moDCs. This procedure was repeated during days 2 and 4 of culture. moDCs with an immature phenotype were harvested on days 5–6 of culture and used for all the assays.

### 4.2. Lyophilization and Protein Quantification

Pulmonarom^®^ is commercially available in 3 mL glass ampoules containing bacterial lysates. A total of 3 mL of Pulmonarom^®^ was lyophilized at −120 °C using a Maxi Dry Lyo system. Protein concentration was then quantified using the Bradford method [64]. The resulting lyophilized powder was stored and reconstituted to the desired concentration prior to use.

### 4.3. moDC Stimulus

The moDCs were stimulated with 0.01, 0.1, and 0.5 µg of Pulmonarom^®^ protein per well with 1 mL of medium supplemented with 10% FBS, 100 U/mL penicillin, 100 µg/mL streptomycin, and 50 µM 2-mercaptoethanol at pH 7.2 in 24 wells of the cell culture plate. They were then incubated for 24 h at 37 °C with 5% CO_2_. Thereafter, supernatants were collected, and the cells were processed for flow cytometry.

### 4.4. Characterization of moDCs

For moDC characterization by flow cytometry, markers evaluating the expression of CD11c, CD1a, HLA, CD80, and CD86 in the flow cytometer (FACS Aria, Becton Dickinson, San Jose, CA, USA) and data were analyzed with the software (FlowJo BD Life Science, V10.8, 2023). The antibodies employed were anti-CD11c (eBioscience, San Diego, CA, USA), CD1a, CD80, HLA, and CD86 (Santa Cruz Biotechnology, Santa Cruz, CA, USA). The DCs harvested on day 7 of culture presented a phenotype, CD11c^+^, CD1a^+^, HLA^lo^, CD80^lo^, and CD86^lo^, corresponding to the phenotype of an immature DC with a purity of ≥80%.

### 4.5. Scanning Transmission Electron Microscopy

Samples were fixed with Karnovsky (2.5% glutaraldehyde/2% formaldehyde) and washed with 0.1M cacodylates. They were postfixed with osmium tetroxide OsO_4_ for 2 h, contracted with 1% uranyl for 1 h, and dehydrated with increasing alcohol concentrations: 70, 80, 90, 100, 100, and 100. Two changes of propylene oxide were added for 30 min each. They were pre-embedded with propylene oxide/Epon resin 2:1 TN, 1:1 TN, and pure TN resin. The inclusion was carried out at 60 °C. Semi-thin sections of 1 micron were made, which were stained with toluidine blue. Thin sections of 100 nm were made and observed in the Crossbeam 550 field emission electron microscope using STEM (Scanning Transmission Electron Microscopy) mode.

### 4.6. Cytokine Production

Cytokines were quantified in cell supernatants after 24 h of stimulation with Pulmonarom^®^ using the Kit Human Anti-Virus Response Panel 1 (Biolegend, Cat. 741270, San Diego, CA, USA) and the Human Essential Immune Response Panel (Biolegend, Cat. 740930) following the supplier’s instructions. Briefly, supernatants were incubated with capture beads for 2 h; thereafter, they were washed with wash buffer (buffer provided in the kit), and then detection biotinylated antibodies were added and incubated for 1 h at room temperature with constant shaking (330 rpm on an orbital shaker). Without washing, streptavidin was added and incubated for another 30 min. Finally, the beads were washed and prepared for the flow cytometer. The limit of quantification (LOQ) for each cytokine, as determined during the calibration procedure of the kit, was as follows: IL-4: 111.42 pg/mL; IL-2: 562.84 pg/mL; IP-10: 65.2 pg/mL; IL-1β: 0.89 pg/mL; TNF-α: 104.06 pg/mL; MCP-1: 43.9 pg/mL; IL-17A: 49.47 pg/mL; IL-6: 33.03 pg/mL; IL-10: 76.32 pg/mL; IFN-γ: 68.64 pg/mL; IL-12p70: 27.7 pg/mL; IL-8: 102.25 pg/mL; IFN-λ1: 249.4 pg/mL; IFN-α: 581.3 pg/mL; IFN-λ2/3: 804.6 pg/mL; GM-CSF: 351.1 pg/mL; and IFN-β: 514.7 pg/mL.

### 4.7. Identification and Expression of Toll-like Receptors (TLRs)

Cells were recovered by cooling the culture plates to 4 °C for 30 min and then washing them with cold phosphate-buffered saline (PBS; NaCl: 137 mM, KCl: 2.7 mM, Na_2_HPO_4_: 10 mM, KH_2_PO_4_: 1.8 mM). The viability and number of the cells were assessed using trypan blue staining. Cells used for flow cytometry assays had a viability greater than 85%. Cells were resuspended in FACS buffer (PBS + 1% bovine serum albumin (BSA)). Cells were adjusted to a concentration of 50,000 cells per tube and stained for flow cytometry assessment. The assay started with viability staining using the Zombie Aqua Fixable Viability Kit (Biolgend Cat. 423102). It was washed with PBS, and then extracellular staining was performed by incubating the antibodies for 30 min with FACS buffer and then washing with PBS. The extracellular antibodies were anti-CD11c-PECy7 (Biolegend Cat. 337216), anti-HLA-DR AF700 (Biolegend Cat. 307626), anti-TLR2 AF 647 (Biolegend Cat. 309714), anti-TLR4 BV 412 (Biolegend Cat. 312811), and anti-TLR6 PE (Biolegend Cat. 334708). For isotype controls, the following antibodies were used: PE Mouse IgG2a k isotype control (BioLegend Cat. 400211) and APC Mouse IgG1 k (Biolgend Cat. 400119).

Once the cells were stained extracellularly, they were fixed with a Fixation Buffer reagent (Biolgend Cat. 420801) for 20 min at room temperature. Thereafter, they were washed with a wash buffer (Biolgend Cat. 421002), diluting the buffer to a 1× concentration. The cells were washed twice by centrifuging at 1500 rpm for 5 min at RT. The cells were incubated overnight (approximately 12 h) with a wash buffer, and the intracellular antibodies used were anti-TLR3 BV711 (Biolgend Cat. 309714), anti-TLR7 FITC (Biolegend Cat. 376908), anti-TLR8 PerCP CY5.5 (Biolegend Cat. 395512), and anti-TLR9 APC- Fire&TRADE 810 (Biolegend Cat. 394812). Finally, the cells were washed with a 1× wash buffer and read by a flow cytometer.

### 4.8. Flow Cytometry Analysis

Samples were read by the NxT Attune flow cytometer, capable of reading 13 colors at a time. Matrix compensation was performed with dendritic cells, and isotype controls were used in each experiment to ensure specific binding to cells. The files obtained were analyzed in FlowJo 10.8 software. Single-parameter analyses were performed using the analysis strategy presented in Supplementary Figure S1 and Figure S2.

### 4.9. Statistical Analysis

The data were first analyzed for distribution using a Shapiro–Wilk test, as appropriate. Data with a normal distribution were analyzed with parametric tests, while data with a non-normal distribution were analyzed with non-parametric tests. In the parametric analysis, the ANOVA–Dunnett test was used to compare the groups. Mean ± standard deviation (SD) was plotted in each graph.

## 5. Conclusions

Pulmonarom^®^ is a safe product currently marketed in Mexico for the treatment of respiratory infections. In this study, we found that Pulmonarom^®^ can modify dendritic cells, and these effects are likely related to the observed increase in the immune response in humans following its treatment.

## Data Availability

The original contributions presented in this study are included in the article/Supplemental Materials. The data that support the findings of this study are available on request from the corresponding author.

## Supplementary Materials

The following supporting information can be downloaded at: https://www.mdpi.com/article/10.3390/ph18060885/s1, Figure S1: Analysis strategy carried out for the identification of dendritic cells from cell cultures. From the CD11c^+^ HLADR^+^ population, the evaluation of TLRs was performed; Figure S2: Analysis strategy for the identification of dendritic cells (CD1a, CD11c, HLADR, CD209) after differentiation; Figure S3: The Pulmonarom^®^ extract does not significantly affect the viability of dendritic cells.
