# Supplementary material for: Immunomodulatory Effects of Pulmonarom®: In Vitro Induction of TLR and Cytokine Expression in Human Dendritic Cells
Source: Pharmaceuticals (Basel). 2025 Jun 13;18(6):885. doi: 10.3390/ph18060885 (PMC12196250; doi:10.3390/ph18060885)
Supplement: Supplementary file 1 [file pharmaceuticals-18-00885-s001.zip › pharmaceuticals-3672001-supplementary.pdf]

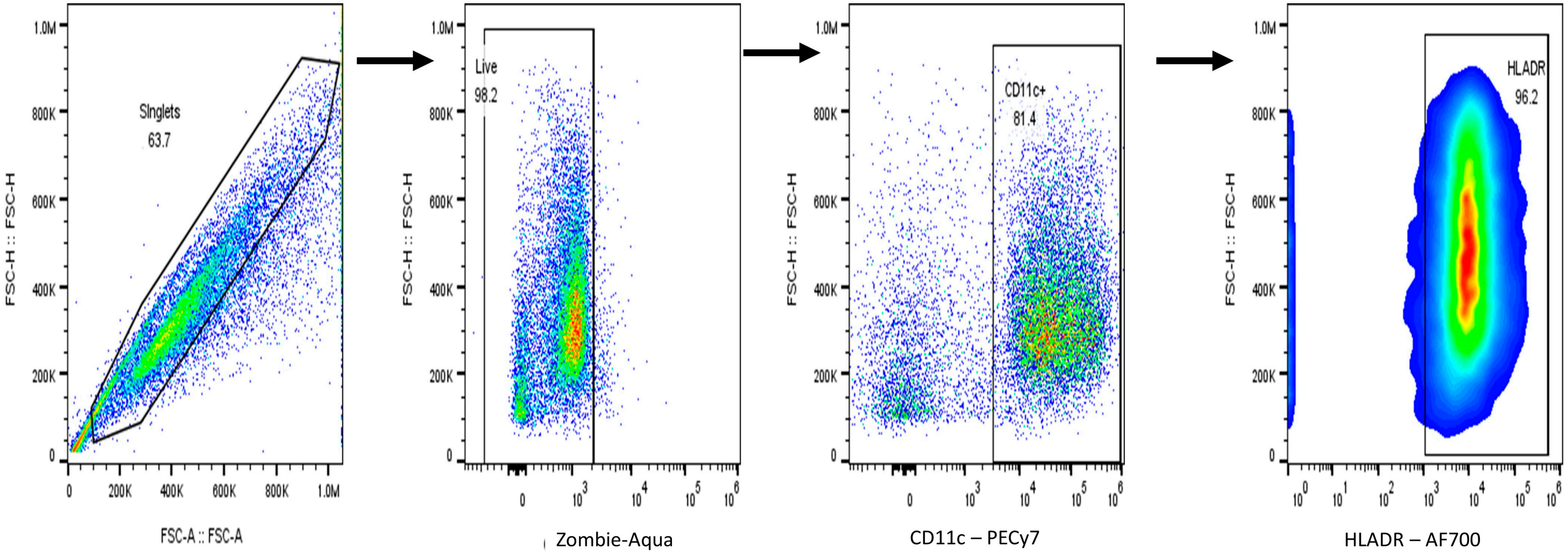

Supplementary Figure S1. Analysis strategy carried out for the identification of dendritic cells from cell cultures. From the CD11c+ HLADR+ population, the evaluation of TLRs was performed.

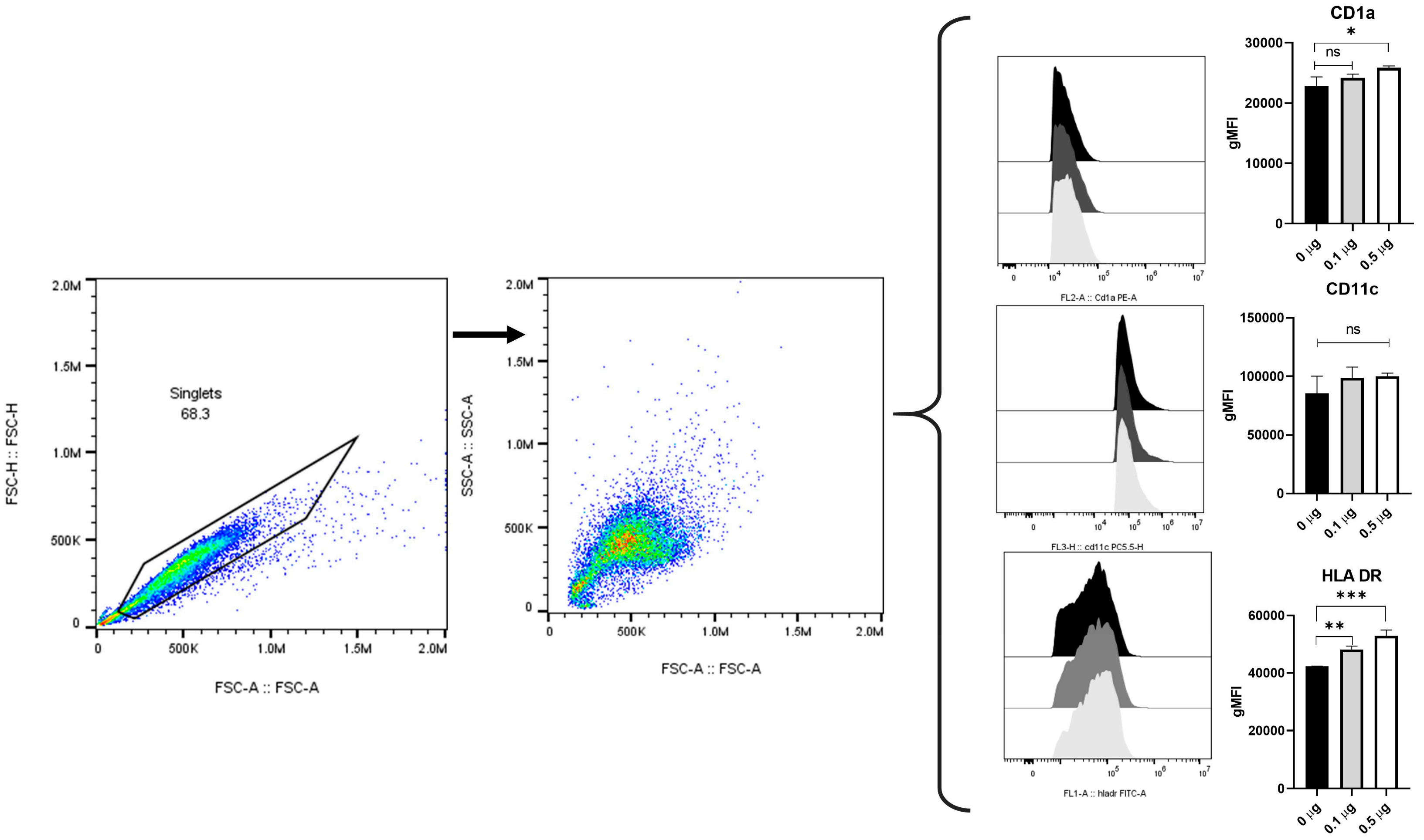

Supplementary Figure S2. Analysis strategy for the identification of dendritic cells (CD1a, CD11c, HLADR, CD209) after differentiation. Furthermore, changes in identification markers in the presence of Pulmonarom® were evaluated. Mean ± standard deviation (SD) was plotted. Data normality was assessed with a Shapiro-Wilks test. ANOVA- Dunnett was used to compare the groups. * p<0.05, ** p <0.01, *** p <0.001, ns: no statistical differences. n=3 per group.

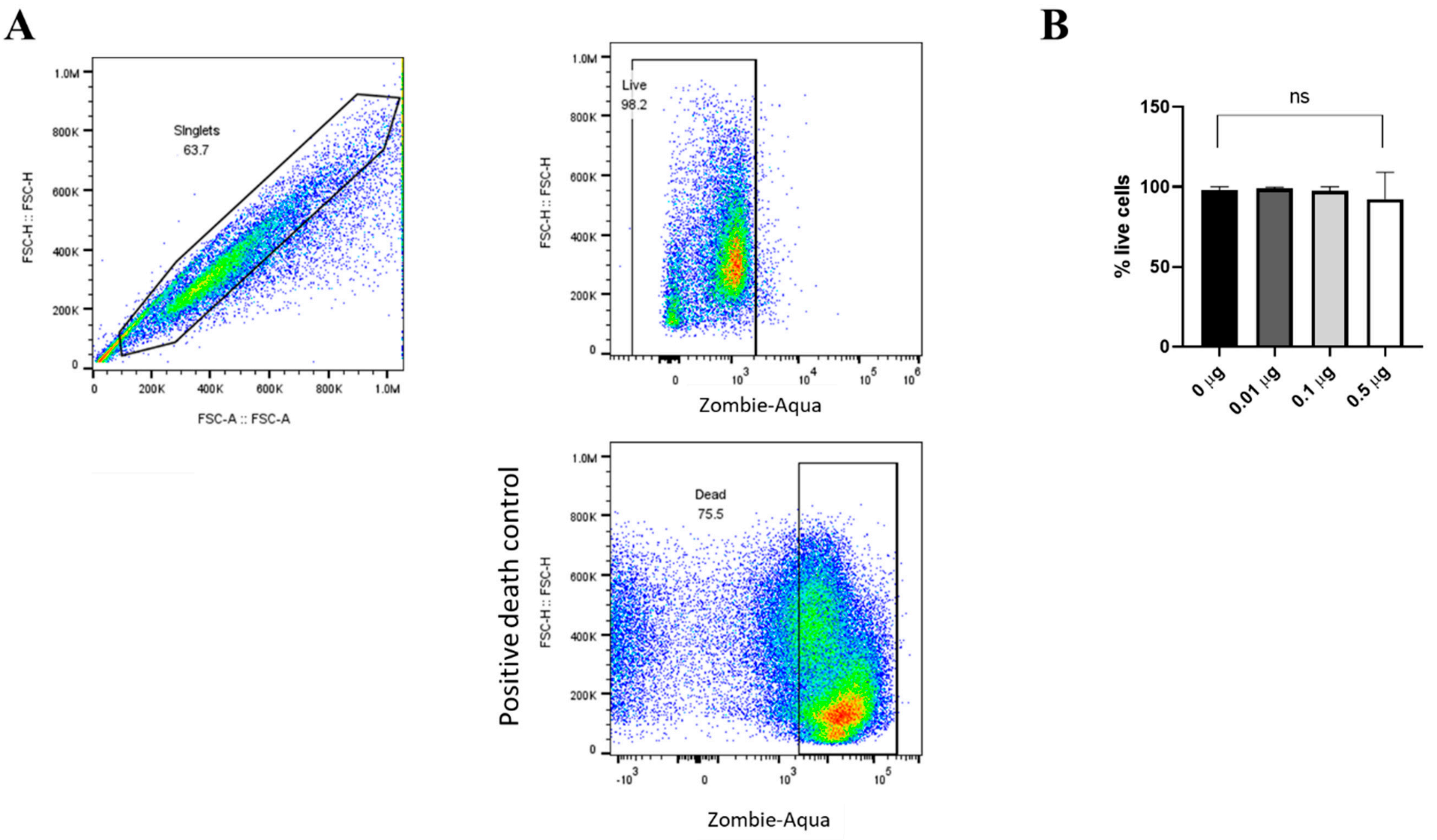

Supplementary Figure S3. The Pulmonarom® extract does not significantly affect the viability of dendritic cells (A,B). Cells detached from cell cultures obtained after stimulation with Pulmonarom® were evaluated. As a positive control for cell death, dendritic cells were incubated in 2 cycles of 30 seconds at 60 °C and 30 seconds at 4 °C, followed by staining with Zombie Aqua to observe dead cells. Mean ± standard deviation (SD) was plotted. Data normality was assessed with a Shapiro-Wilks test. ANOVA-Dunnett was used to compare the groups., ns: no statistical differences. n=5 per group.
